# Efficacy of 1064 nm Photobiomodulation Dosimetry Delivered with a Collimated Flat-Top Handpiece in the Management of Peripheral Facial Paralysis in Patients Unresponsive to Standard Treatment Care: A Case Series

**DOI:** 10.3390/jcm12196294

**Published:** 2023-09-29

**Authors:** Sonja Zarkovic Gjurin, Jason Pang, Mihael Vrčkovnik, Reem Hanna

**Affiliations:** 1 Department of Prosthodontics, Faculty of Medicine, University of Ljubljana, SI-1000 Ljubljana, Slovenia; sonja.zarkovic@mf.uni-lj.si; 2 Gemelli University Hospital, Università Cattolica del Sacro Cuore, 00168 Rome, Italy; 3 Cosmic Smile Laser Dental, Sydney, NSW 2089, Australia; 4 Laserski Center MV, 1000 Ljubljana, Slovenia; info@laserskicenter.si; 5 Department of Oral Surgery, King’s College Hospital NNS Foundation Trust, London SE5 9RS, UK; 6 Department of Restorative Dental Sciences, UCL-Eastman Dental Institute, Medical Faculty, University College London, London WC1E 6DE, UK; 7 Department of Surgical Sciences and Integrated Diagnostics, University of Genoa, 16126 Genoa, Italy

**Keywords:** neurological disorders, oxidative stress, Lyme disease, Bell’s palsy, Ramsay Hunt syndrome, pulsed Nd:YAG, photobiomodulation, peripheral facial paralysis, facial exercises, flat-top beam profile

## Abstract

Peripheral facial paralysis (PFP) is a common condition where oxidative stress (OS) is involved in the pathophysiology of facial paralysis, inhibiting peripheral nerve regeneration, which can be featured in Bell’s palsy, Ramsay Hunt syndrome and Lyme disease. The current standard care treatments lack consensus and clear guidelines. Hence, the utilization of the antioxidant immunomodulator photobiomodulation (PBM) can optimize clinical outcomes in patients who are unresponsive to standard care treatments. Our study describes three unique cases of chronic PFP of various origins that were unresponsive to standard care treatments, but achieved a significant and complete recovery of facial paralysis following PBM therapy. Case presentations: Case #1: a 30-year-old male who presented with a history of 12 years of left-side facial paralysis and tingling as a result of Bell’s palsy, where all the standard care treatments failed to restore the facial muscles’ paralysis. Eleven trigger and affected points were irradiated with 1064 nm with an irradiance of ~0.5 W/cm^2^ delivered with a collimated prototype flat-top (6 cm^2^) in a pulsed mode, with a 100 µs pulse duration at a frequency of 10 Hz for 60 s (s) per point. Each point received a fluence of 30 J/cm^2^ according to the following treatment protocol: three times a week for the first three months, then twice a week for another three weeks, and finally once a week for the following three months. The results showed an improvement in facial muscles’ functionality (FMF) by week two, whereas significant improvement was observed after 11 weeks of PBM, after which the House–Brackmann grading scale (HBGS) of facial nerve palsy dropped to 8 from 13 prior to the treatment. Six months after PBM commencement, electromyography (EMG) showed sustainability of the FMF. Case #2: A five-year-old female who presented with a 6-month history of severe facial paralysis due to Lyme disease. The same PBM parameters were utilized, but the treatment protocol was as follows: three times a week for one month (12 consecutive treatment sessions), then the patient received seven more sessions twice a week. During the same time period, the physiotherapy of the face muscles was also delivered intensively twice a week (10 consecutive treatments in five weeks). Significant improvements in FMF and sustainability over a 6-month follow-up were observed. Case #3: A 52-year-old male who presented with severe facial palsy (Grade 6 on HBGS) and was diagnosed with Ramsay Hunt syndrome. The same laser parameters were employed, but the treatment protocol was as follows: three times a week for three weeks, then reduced to twice a week for another three weeks, then weekly for the next three months. By week 12, the patient showed a significant FMF improvement, and by week 20, complete FMF had been restored. Our results, for the first time, showed pulsed 1064 nm PBM delivered with a flat-top handpiece protocol is a valid and its treatment protocol modified, depending on the origin and severity of the condition, which is fundamental in optimizing facial paralysis recovery and alleviating neurological symptoms. Further extensive studies with large data are warranted to validate our PBM dosimetry and treatment protocols.

## 1. Introduction

Idiopathic peripheral facial paralysis is a common condition with an annual incidence between 15 and 30 cases per 100,000 inhabitants [1].

### 1.1. Role of Oxidative Stress in Pathogenesis of Peripheral Facial Paralysis (PFP)

Mitochondria are recognized as a key regulator of cellular function, especially in stressful and viral events triggering alteration of energetic and metabolic homeostasis of mitochondrial function [2]. Oxidative stress (OS) is one of them. It is associated with the imbalance between reactive oxygen species (ROS) and the antioxidant defense system, resulting in the pathogenesis of various diseases, including neurodegenerative disorders, presenting a crosslink between OS and inflammation [1]. 

It is noteworthy that a neuron cell relies heavily on mitochondrial oxidative phosphorylation to generate an extensive volume of adenosine triphosphate (ATP) that is required for daily cell needs. In PFP, the facial nerve, which is the 7th cranial nerve, is affected [2]. An in vitro study conducted by Persson et al., 2012 utilizing model-induced axonal injury showed that sodium channels contributed to degeneration of the dorsal root ganglion neurites induced by mitochondrial dysfunction [3]. 

### 1.2. Various Diseases Related to PFP and Current Standard Treatment Care

Currently, there is neither consensus nor clear guidelines on the optimal ideal standard treatment care for PFP; however, the following treatment modalities have been employed: pharmacotherapy (antiviral, corticosteroid and analgesic) [4,5], electrotherapy, biofeedback, facial massage, physical exercises, physiotherapy [6,7], acupuncture, electrical stimulation, botulinum toxin [8] and photobiomodulation [1,9,10,11].

An overview and description of the neurogenerative diseases presented in our unique case series of three very distinct cases of PFP of different origins and a wide age range are described below.

#### 1.2.1. Bell’s Palsy 

Bell’s palsy (BP) is the most common facial paralysis, with an incidence between 60 and 75% of all the cases. It affects the 7th cranial nerve and is presented as acute, with unilateral onset. It is associated with aesthetical and functional disabilities, which subsequently have a great impact on the social, professional and psychological aspects of the affected individuals [9]. 

Even though idiopathic, viral/bacterial infection, neoplastic, toxic exposures, vascular ischemia, micro-trauma, autoimmune or even genetic reasons can be counted as responsible [10,11], reactivation of the herpes simplex virus-1 (HSV-1) at the cranial nerve is suspected to be the most common cause of facial nerve inflammation in BP [10]. This inflammation causes compression of the 7th cranial nerve at the geniculate ganglion and, subsequently, ischemia of the nerve.

BP has an annual incidence between 23 and 35 cases per 100,000 inhabitants, with no significant difference between genders [10,11]. Both sides of the face are equally affected, with less than 1% of cases being bilateral [11]. In terms of BP recovery, it usually begins at 2 weeks up to 6 months from the initial symptoms’ onset. Most patients with BP can gain recovery of full facial strength and expressions [12].

#### 1.2.2. Lyme Disease

Lyme borreliosis is a common tick-borne disease in the northern hemisphere. It is caused by spirochaetes of the *Borrelia burgdorferi sensu lato* (*B. burgdorferi s. l.*) complex. Typically, at first, the disease presents with an erythema migrans rash at the site of the tick bite, followed by flu-like symptoms and later by debilitating arthritic, dermatological and neurological manifestations [13].

Lyme disease (LD) in children presents itself with neurological manifestations mostly associated with facial nerve (7th cranial nerve) palsy and lymphocytic meningitis [14], leading to PFP due to swelling and impingement of the facial nerve, as it tracks through the narrow bony fallopian canal in the skull beneath the ear [15]. The prognosis of neurological LD in children is excellent if an early diagnosis and an appropriate therapy are employed [14].

#### 1.2.3. Ramsay Hunt Syndrome

Postherpetic neuralgia (PHN) is the most common chronic complication of varicella-zoster virus (VZV) reactivation. The incidence of PHN is around 18% at 50 years of age, whereas the percentage of patients who experience PHN for longer than one year is between 30% and 50%, resulting in cranial nerve dysfunction, orbital movement impairment, facial hypoesthesia, cochleo-vestibular symptoms, dysphagia and vocal paralysis [16]. There is no single effective treatment that can reduce PHN incidence [17]. PFP is one symptom of Ramsay Hunt syndrome (RHS) that occurs by reactivation of VZV in the geniculate or facial nerves [18]. Due to anatomical association with other cranial or cervical spinal nerves, various clinical features can present, such as tinnitus, hearing loss, nausea, vomiting, vertigo and nystagmus, resulting in permanent hearing loss, facial weakness, eye damage and postherpetic neuralgia [19].

### 1.3. Photobiomodulation Contributions an Immunomodulator in PFP

**Figure 1:**
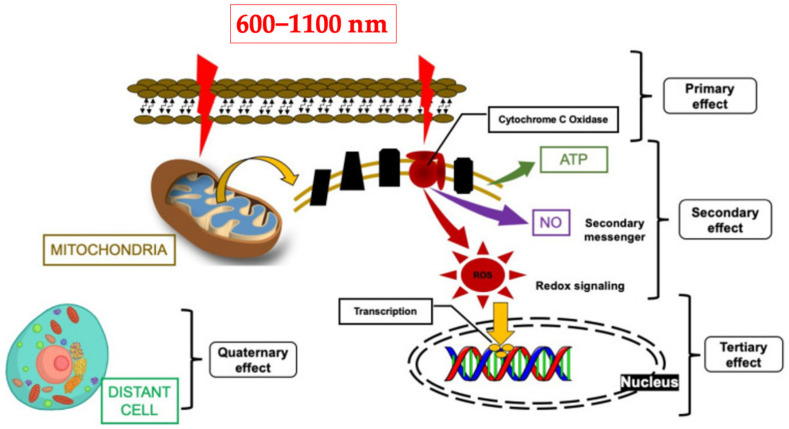
Shows the current mechanism of action of photobiomodulation therapy. (Permission obtained from Hanna et al., 2021 [21]).

Figure 1 shows that the mechanism of action of PBM seems to operate at local, regional and systemic levels. Red and near-infrared (NIR) photobiomodulation (PBM) can restore mitochondrial homeostasis, where its photonic energy is absorbed by cytochrome c oxidase [(CCO), photoacceptor] in the mitochondrial respiratory chain [20], resulting in a cascade of cellular and molecular activities such as ATP synthesis and modulating nitric oxide (NO) and ROS [20,21,22] (Figure 1). An increase in intracellular ATP can alter the functions of cellular metabolism and is one of the most frequent and significant findings after PBM in vitro and in vivo [23,24]. 

Photo-induced ROS response can be associated with laser-induced analgesia [25,26,27]. PBM also inhibits cyclooxygenase, which results in the reduced production of prostaglandins and thus reduces the acute inflammatory response [28]. A review conducted by Hanna et al., 2021 showed the efficacy of PBM as an immunomodulator and anti-inflammatory in the management of potentially malignant oral lesions [21]. Also, many clinical studies [29,30] and reviews [31,32] have shown PBM efficacy as antiviral and immunomodulatory in reducing viral load and minimizing hospitalization in patients with COVID-19 and in restoring smell, taste and fog in patients with long COVD-19 sequelae.

Evidence-based science and practice reported that PBM is a safe, non-invasive, immunomodulatory therapy to alleviate pain [25,26,27], reduce inflammation [33] and enhance tissue repair and regeneration [34,35,36], which ultimately promotes positive tissue processes. 

### 1.4. Properties of Collimated Flat-Top Beam Profile

To achieve standardization and reproducibility of PBM dosimetry, a collimated flat-top beam profile was employed. The flat-top beam profile allowed a static approach to treatment for consistent dosimetry and depth penetration as compared to a gaussian beam profile, which may have difficulty achieving consistent outcomes, optical delivery to target depth, as well as calculation of dosimetry [35,37,38,39]. This is related to the unique property that the collimated flat-top, offering equal and uniform distribution of the photonic energy over more than 1 cm^2^ compared with a gaussian beam profile, offering its maximum energy only at the center of the beam [37]. Hence, in the present study, we utilized a collimated flat-top beam profile to deliver an equal distribution of 1064 nm PBM photonic energy over a large target surface area.

### 1.5. Rationale in Conducting the Present Case Series Study

Recent systematic reviews and meta-analyses have validated that PBM as a noninvasive, antioxidant and regenerative therapy can play an influential role in the management of various clinical applications such as oral lichen planus, recurrent aphthous stomatitis, hyposalivation, pemphigus vulgaris, recurrent herpes simplex, burning mouth syndrome, oral mucositis, medications-related to osteoradionecrosis of the jaws, trigeminal neuralgia and facial nerve paralysis [21,22,25,26,27,40,41]. Therefore, they concluded that PBM therapy can be effective as an alternative treatment or in combination with other therapies in improving symptoms or in the complete treatment of oral diseases, but further clinical studies are still necessary to achieve more robust results [42]. Moreover, no single effective treatment reduced PHN incidence; however, it has been reported that photobiomodulation (PBM) was able to reduce the duration and intensity of pain in many reports [43]. Laser PBM has been reported to cause significant improvement in facial movement compared to exercise therapy alone [44]. Hence, the present case series study aimed to assess the effectiveness of laser PBM therapy in relieving facial paralysis in three very different cases of PFP. The first case describes idiopathic facial palsy in an adult male; the second case reports on face palsy due to Lyme disease in a child; and the last one reports on RHS.

## 2. Materials and Methods

### Study Design

A case series of three unique, moderate-to-severe cases of chronic PFP of various origins in which patients failed to respond to the standard care treatment. The three cases were as follows: Bell’s palsy (BP), Lyme disease (LD) and Ramsay Hunt syndrome (RHS). All the cases received photobiomodulation therapy with different treatment durations, depending on the patient’s response. An informed written consent was obtained from all the patients and legal guardians, and a full explanation about the treatment was given, including a patient information leaflet. Also, written informed consent from all the subjects was obtained to publish their facial photos, which might reveal their identity (essential for treatment prognosis) when publishing this paper in the journal. The study was conducted in accordance with the Declaration of Helsinki. 

The variables’ progression for all the cases was assessed using the House–Brackmann grading scale (HBGS) and the patient’s self-reporting assessment. HBGS is utilized as an assessment tool to rank facial muscles’ weakness and movement impairments associated with BP, LD and RHS. It is a valid and reliable tool to assess the overall and segmental muscle weakness in the face [45]. The facial movement impairment grade ranges between I and VI, where grade I indicates normal facial muscle contraction and grade VI indicates no possible movement [45].

The laser device that was employed for all the cases was a solid-state free-running-pulse Nd:YAG (1064 nm) laser (LightWalker ATS^®^; Fotona, Ljubljana, Slovenia), where its aiming beam was from a low-power visible green semiconductor diode laser (532 nm, power < 1 mW) and was transmitted coaxially along the optical fiber. The delivery system was a flat-top collimated probe with the following unique specifications: (1) emits an energy beam covering an area of 6 cm^2^, (2) provides the same power density over any tip-to-tissue distance from contact up to around one meter, and (3) provides uniform distribution of the photonic energy over the target surface area [37]. The collimated prototype flat-top beam profile targets a large surface area of 6 cm^2^ (Fotona, Ljubljana, Slovenia). The laser device and the flat-top delivery device were purchased by the investigator, who has a depth of experience in utilizing PBM therapy.

The therapeutic PBM parameters were confirmed with the PM160T power meter, Thorlabs, Newton, NJ, USA. Any possible undesirable thermal effects were avoided by monitoring PBM irradiation with a thermal camera FLIR ONE Pro-iOS (FLIR Systems, Inc. designs, Portland, OR, USA).

The laser PBM dosimetry protocol that was employed for all three cases was as follows: λ: 1064 nm (LightWalker ATS, Fotona LLC, Ljubljana, Slovenia); therapeutic power output: 0.5 W/cm^2^; frequency: 10 Hz; pulse width: 100 µs (MSP); beam profile: prototype flat-top collimated with a surface area of 6 cm^2^; irradiation time: 60 s per spot; fluence: 30 J/cm^2^ per spot (Table 1).

## 3. Case Presentations

### 3.1. Case 1: Facial Paralysis Related to Bell’s Palsy

#### 3.1.1. Initial Clinical Presentation 

A 30-year-old male presented with a history of 12 years of left-side idiopathic facial paralysis and tingling as a result of Bell’s palsy (BP). He initially woke up with weakness in all the muscles of his left facial expression. He experienced the following symptoms: an inability to raise the left corner of his mouth while smiling; a lack of ability to contract his left side of his forehead and raise his eyebrows; a strange taste in his mouth and weakness in the movement of the left side of his tongue; he could not close his left eye, but tactile feeling was minimally changed. The laboratory investigation for testing for borreliosis was negative. Subsequently, the patient was diagnosed with idiopathic paralysis of the left facial nerve. 

#### 3.1.2. Initial Management Prior to PBM Therapy

Eye drops were prescribed, and eye protection was provided during the night. The patient was also advised to use vitamin A eye gel. Facial exercises were prescribed. Spontaneous remission was expected in a few weeks; however, there were no changes in the patient’s symptoms. 

Ten months after the initial presentation of facial paralysis, a neurologist measured the M wave with stimulation of the left facial nerve. The amplitude was half that of the right side of the face.

A year after the condition remained persistent, electro-stimulation therapy of the damaged area was performed. Ongoing action potentials were detected even during “the resting phase” with electromyography (EMG). In all of the examined muscles, weakness and chronic neuropathic changes were observed. In the forehead muscle, the innervation was good but severely limited in the other facial muscles.

**Figure 2:**
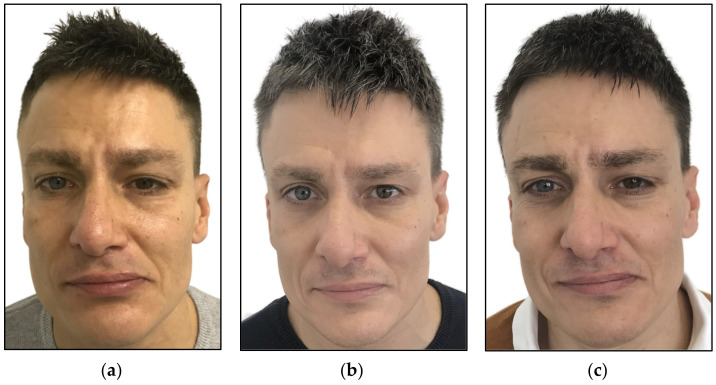
(**a**) Shows a lack of left facial muscles’ functionality; (**b**) shows no improvement after nine weeks of PBM therapy; (**c**) shows a significant improvement in the symptoms, smiling better and opening his left eye much better after 11 weeks. Despite the persistence of facial asymmetry, there was a significant improvement in facial functionality.

One year later, the functionality of the tongue muscles recovered, but the left facial muscles’ weakness remained. Additionally, the patient reported that his left facial muscles were stiffer than at the beginning of the initial symptoms of paralysis. Further neurological examination determined practically total axonal damage of the left facial nerve, which was severely damaged in the lower half of the face, causing a severe asymmetry, and was considered most likely permanent. Despite physiotherapy sessions and the patient’s daily physical exercise in front of a mirror, no symptoms’ improvement was achieved. An absence of a neurophysiological response was indicative of complete nerve degeneration and prolonged paralysis with incomplete recovery that may be complicated by synkinesis [46]. Hence, the patient was referred to our Laser Center for PBM therapy (Figure 2a).

#### 3.1.3. PBM Treatment Protocol

Initially, the treatment frequency was three times a week for three consecutive weeks. Subsequently, it was reduced to twice a week for another three consecutive weeks. Then, it was reduced to weekly for the following three months. The total number of irradiated spots per session was seven (Table 2). 

#### 3.1.4. Results of PBM Therapy

During the first week of PBM treatment, no improvement was observed, whereas after 9 weeks of PBM, changes in the cheek tingling sensation were noticed (Figure 2b).

After 11 weeks, there was a significant turning point. The functionality of the muscles around the lip (orbicularis oris and buccinator muscles) improved, and the patient was able to whistle and smile much better. The patient reported that the facial skin was less stiff compared to pretreatment. This was indicative of an improvement in the relaxation of the muscles relating to facial expressions. An improvement in the smell sense was reported. Also, the patient was able to open his left eye wider. Despite the patient’s incomplete facial symmetry, a major improvement was observed (Figure 2c) compared to pretreatment (Figure 2a). The patient continued to improve in his symptoms, and at 6 months after the PBM therapy was completed, a significant improvement in facial muscles’ functionality was confirmed by EMG recordings compared to the EMG findings prior to PBM treatment. All those findings were reported by the same neurologist.

**Figure 3:**
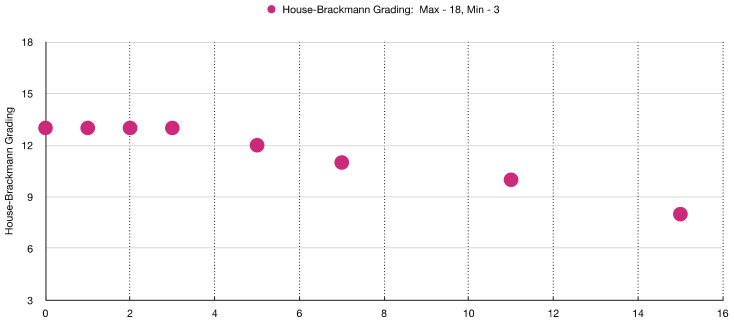
Shows a significant improvement of the facial muscles’ functionality on the House–Brackmann grading scale over the course of PBM therapy.

Table 3 and Figure 3 show the sequence of facial muscles’ improvements over the timepoints of 15 weeks of PBM treatment evaluated with HBGS.

### 3.2. Case 2: Facial Palsy Related to Lyme Disease 

#### 3.2.1. Initial Clinical Presentation

A five and a half-year-old child developed a high fever (40 °C) in the summer of 2020 associated with headaches and pain in her leg. She was examined by her pediatrician twice, and her C-reactive protein level was 50 mg/L, indicating systemic inflammation. Two weeks later, right facial asymmetry with redness (red lines) on the right side of the face was noticed, and the patient was unable to close her eyelids. The patient was under the care of her pediatrician, who diagnosed her with peripheral paralysis of the facial nerve (Grade 6 on HBGS). The results of the blood workup and serology analysis confirmed the presence of *Borrelia burgdorferi*, and the child was diagnosed with neuroborreliosis with facial paralysis. 

#### 3.2.2. Initial Management Prior to PBM

The first line of treatment was as follows: a combination of Ceftriaxone for ten days and physical therapy three times a week; application of vitamin cream to conjunctiva daily, as well as artificial tear drops; oral vitamin B complex. A month later, the patient presented with the following persistent symptoms: severe facial asymmetry, difficulty closing her eyelids completely (they remained 5 mm open), minimal contraction and movement of her eyebrow, and drooping of the left corner of her mouth. This indicated that this treatment regime was unsuccessful. Hence, the patient was referred to our laser center for photobiomodulation therapy. Two weeks later, laser PBM therapy commenced.

#### 3.2.3. PBM Treatment Protocol

**Figure 4:**
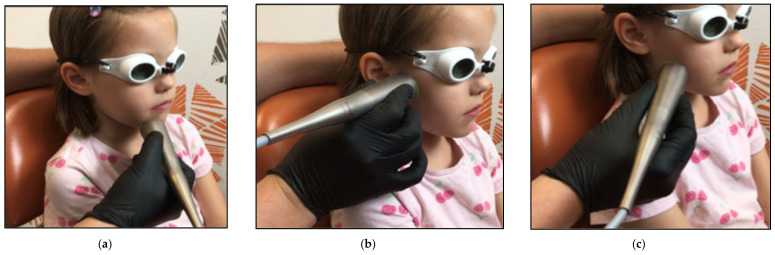
Shows the applications of 1064 nm laser PBM delivered with a flat-top collimated handpiece, targeting the branches of the 7th cranial nerve (facial nerve) to treat the patient with Lyme disease palsy. (**a**) the application of the flat-top handpiece targeting the marginal mandibular nerve branches; (**b**) the application of the flat-top handpiece targeting the zygomatic branches; (**c**) the application of the flat-top handpiece targeting buccal branches.

In the first month of commencing PBM therapy, the patient received 12 consecutive sessions three times a week, and at the same time, physiotherapy of the facial muscles was delivered intensively twice a week (10 consecutive therapies in five weeks). Then the patient received another seven sessions twice a week. The number of irradiated spots was 12 (Table 4). Figure 4 shows the applications of the flat-top collimated handpiece on the irradiated spots along the distribution of the facial nerve branches. The handpiece was held perpendicular to the facial surface area and in contact with the skin.

#### 3.2.4. Results after PBM Therapy and Physiotherapy

**Figure 5:**
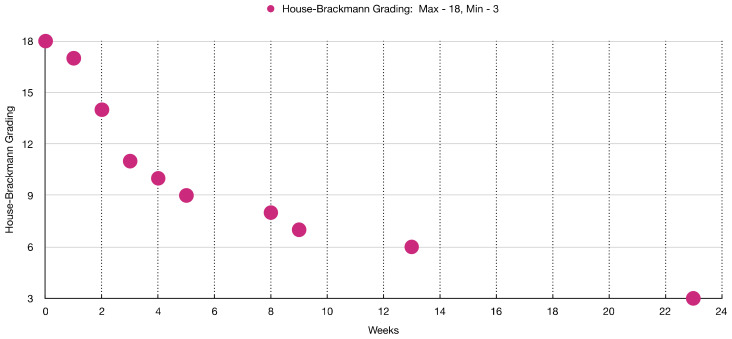
Shows complete recovery of the facial muscles’ functionality on the House–Brackmann Grading Scale over the course of PBM therapy.

After the first two PBM sessions, the shadow above the eyebrow was noticed when the patient was trying to move her eyebrow. Whereas after five sessions of PBM therapy, an improvement in the muscles in the right corner of the mouth was noticed by the patient’s mother and the rest of the family, as well as the kindergarten teacher. A significant improvement in controlling the right facial movements was observed between the 7th and 11th PBM sessions. Whereas, the corner of the mouth was slightly turned up after the 9th session of PBM (Figure 5).

After twelve consecutive PBM sessions and ten sessions of physiotherapy, a significant improvement in the movement of the right facial muscles and in facial asymmetry was observed. A month after that, an additional seven PBM sessions without physiotherapy were delivered to enhance the symptoms further. At the 15th PBM session, there was a significant improvement in the functionality of the muscles of the right eyelid and facial symmetry. Two weeks after completing PBM sessions, the patient was able to completely close her eyelid. Subsequently, after completing the PBM therapy, an additional ten sessions of physiotherapy were delivered. Despite the physiotherapy, the deficit on the upper lip and lateral aspect of the nose was persistent.

**Figure 6:**
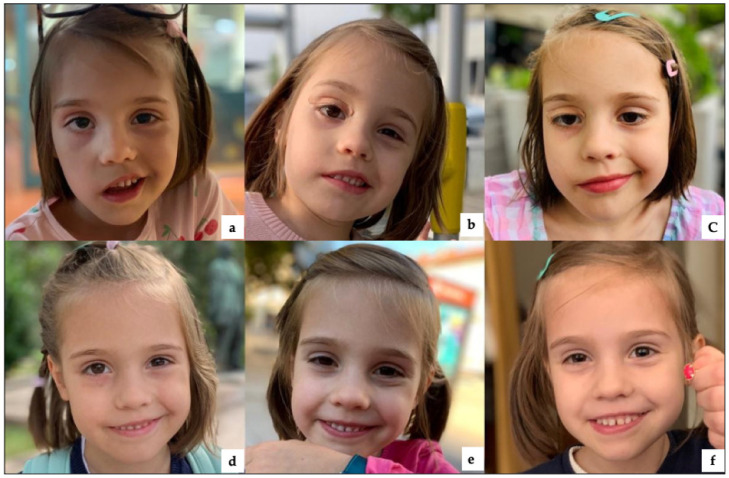
Shows an improvement of the right facial muscles of the patient with Lyme disease palsy after receiving the following therapies: only PBM, combined PBM and physiotherapy, only physiotherapy, and only PBM sessions. (**a**) day zero; (**b**) after one week of PBM, no improvement was observed; (**c**) after two weeks of PBM, the patient felt a movement in the right side of her face and was also noticed by her parents and others (kindergarten teachers); (**d**) 4 weeks after PBM, the patient’s facial movements improved; the eyelid almost closed and the right mouth angle was turning up; (**e**) 8 weeks post-PBM therapy, the child felt a great improvement in her facial muscles. The eyelid was still not completely closing during the night; (**f**) 10 weeks post-PBM therapy, the patient felt very well in her facial muscles’ movements; the eyelids closed symmetrically as well at night.

Five months after the last session of PBM, a decision was made with the consent of the patient’s mother to deliver additional PBM sessions to restore the buccal branches of the right facial nerve that are responsible for the muscles of the lateral nose, nasolabial fold and upper lip. Figure 5 and Figure 6 show the sequence of PBM therapy timepoints and their relation to the patient’s improvement in facial paralysis.

Table 5 shows in detail the level of facial muscles’ functionality and facial symmetry improvement over the course of PBM therapy compared to prior to treatment at week “0”, where the total value at HBGS was “18” and at week 23 of PBM, the total HBGS of symptoms was “3”. Significant improvement led to achieving facial symmetry and normal facial muscles’ functionality. 

### 3.3. Case 3: Ramsay Hunt Syndrome

#### 3.3.1. Clinical Presentation and Initial Management

**Figure 7:**
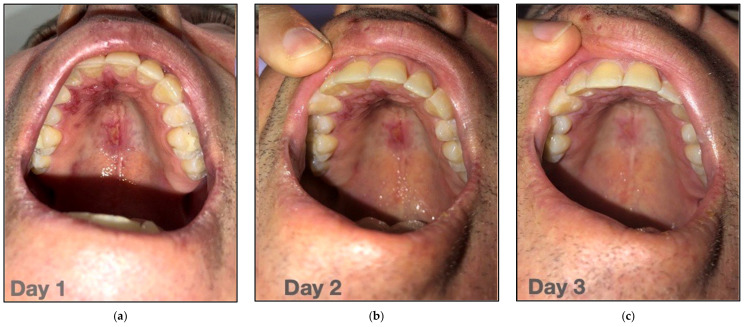
Herpetic lesions on the upper lip and palate initially appeared prior to the development of FNP (**a**) day one, (**b**) day two, (**c**) day three.

A 52-year-old male presented with severe facial nerve paralysis (FNP) on the right side of the face. It was Grade 6 paralysis on the HBGS. He was diagnosed with RHS by a neurologist as a result of a Herpes Zoster infection of the 7th facial nerve (CN VII). Painful herpetic lesions appeared on the right upper lip and in the palate prior to the development of FNP (Figure 7). The patient started to experience pain around the right ear along with a mild facial rash on Day 5. The area of pain extended backward from the right ear to the occiput (lower and back parts of the cranium).

The facial palsy and taste loss commenced on Day 9 when the patient presented to his local hospital. He was prescribed a high dose of Valaciclovir, 1 g, three times a day (TDS), for a week, and Prednisolone, 120 mg, for 2 days, with the initial dose tapering over two weeks. The patient did not experience any hearing or balance disturbances. In the meantime, he was referred to a neurologist, where he was diagnosed with RHS. The patient felt no improvement after the completion of the above treatment regime.

#### 3.3.2. Clinical Presentation Prior to PBM Therapy

**Figure 8:**
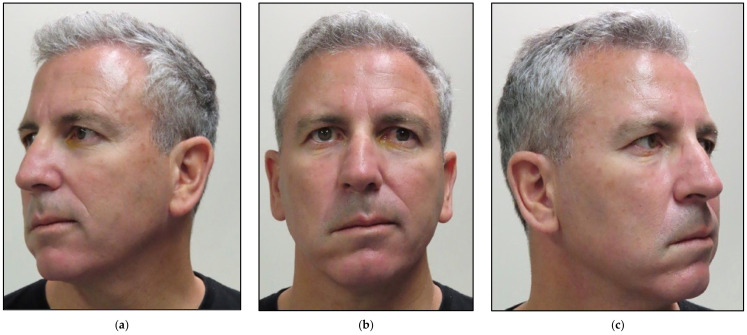
Shows patient’s presentation prior to the initial presentation with facial palsy on the right-hand side of the face. (**a**) lateral view shows difficulty in smiling and making facial movements; (**b**) frontal view shows facial asymmetry related to paralysis of the right side of the face; (**c**) lateral view shows drooping of the right corner of the mouth and smoothing of the right forehead.

The patient was referred to our laser center and presented on Day 18, where no oral lesions were noted. At presentation, the patient was experiencing sporadic, spontaneous, burning pain around an occiput of 8/10 on VAS. His sleep was disturbed, and he managed to get only 2 h of sleep per night. He had difficulty controlling the right side of his tongue; hence, he had poor clearance of food during eating due to the lack of tongue movements. Additionally, there was a lack of taste and partial numbness in his right lateral tongue. There were no dentoalveolar complications to report. His hearing and balance were normal. The patient presented with facial asymmetry, where all the branches of the facial nerve were affected (Figure 8).

#### 3.3.3. PBM Dosimetry and Treatment Protocols

We utilized the same PBM laser dosimetry protocol and device specifications that we employed in case #1 (Table 1), whereas the treatment protocol and number of irradiated points were as follows, respectively: three times a week for three weeks. Subsequently, this was reduced to twice a week for another three weeks and then weekly for the next three months. The number of irradiated spots was eleven (Table 6). The true PBM emission parameters were confirmed using a power meter.

**Figure 9:**
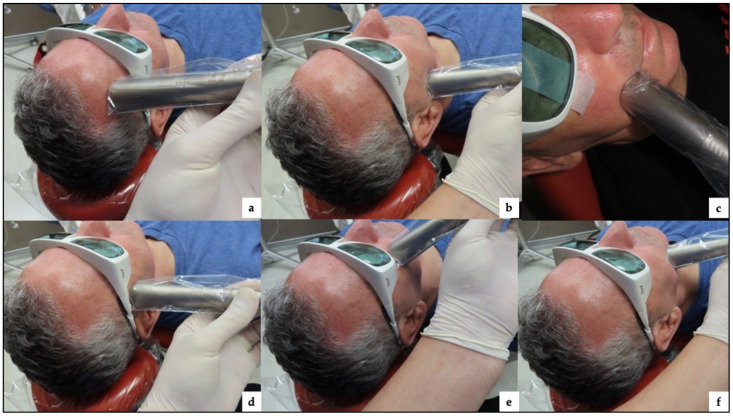
Shows the application of the photonic energy of 1064 nm laser PBM delivered with a collimated flat-top handpiece along the distribution of the facial nerve branches innervating the following muscles: (**a**) frontalis; (**b**) buccinator muscle; (**c**) orbicularis oris; (**d**) zygomatic muscle; (**e**) Levator anguli oris; (**f**) depressor anguli oris.

Figure 9 shows the application of a flat-top handpiece to irradiate the spots along the distribution of the affected facial nerve branches.

#### 3.3.4. Results after PBM Therapy

Table 7 illustrates the relationship between the PBM course of treatment and facial muscles’ functionality and facial symmetry evaluated with the HBGS. The symptoms’ progression over the PBM treatment timepoints is explained below:

1. During the first week, the patient required less analgesic during the day and was able to progressively sleep more each night. Only slight movement improvement was observed in the eye, mouth and forehead.
2. At week 2, more mouth and forehead movements were observed, but little additional eye movement. Longer sleep periods were observed, and pain was intermittent.
3. At week 3, good improvement in the movement of the forehead muscles was observed, but there was only marginal improvement in the eye and mouth movements.
4. At week 4, the patient was able to have 8 h of uninterrupted sleep due to only a slight neuralgic pain. Additionally, there was more eyebrow movement and tingling at the corner of the mouth and the chin, with some improvement in function.
5. At week 6, there was no more neurological pain, which resulted in restful sleep. Lip function had improved, and the patient was able to whistle and eat comfortably. The forehead movement was good, and the patient was able to close his eye voluntarily.
6. At week 8, the patient’s face was much more symmetrical, with the eye slowest to improve. The blink reflex was better, and the forehead muscles were moving very well, but there was still numbness and poor tone in the upper lip.
7. At week 12, the patient’s smile was almost symmetrical. The blink motion was much faster, and he was feeling mentally and physically stronger. He continued to have restful sleep with no neuralgia.
8. At week 20, the subject’s eye and mouth had almost complete movement; his face was symmetrical, and no further treatment was required.

**Table 7:** Shows an improvement of Ramsay Hunt symptoms over the PBM course of treatment using the House–Brackmann grading scale (HBGS).

PBM Treatment Week Number	Functionality Level	Facial Symmetry	Facial Muscles’ Functionality			Total
			Forehead	Eye	Mouth	
0	Severe	Asymmetric	6	6	5	17
1	Severe	Asymmetric	5	5	5	15
2	Moderately severe	Asymmetric	4	5	4	13
3	Moderate	Asymmetric	3	4	4	11
4	Moderate	Asymmetric	3	4	3	10
5	Moderate	Asymmetric	3	3	3	9
6	Moderate	Asymmetric	2	3	3	8
8	Mild	Mildly asymmetric	2	3	2	7
12	Mild	Symmetric	1	3	2	6
20	Almost normal	Symmetric	1	2	2	5

**Figure 10:**
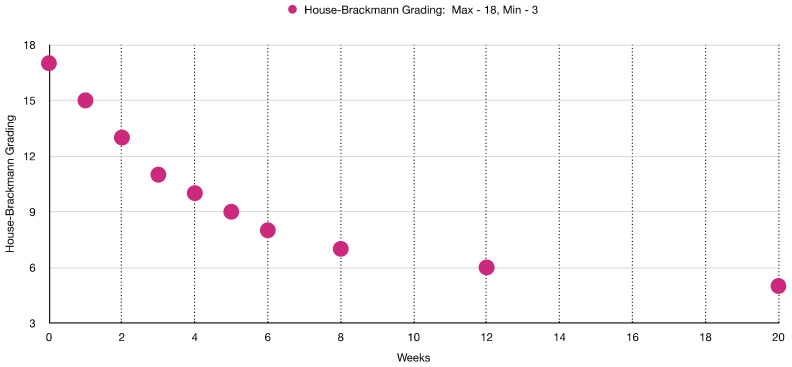
Shows almost complete recovery of the facial muscles’ functionality on the House–Brackmann grading scale over the course of PBM therapy.

**Figure 11:**
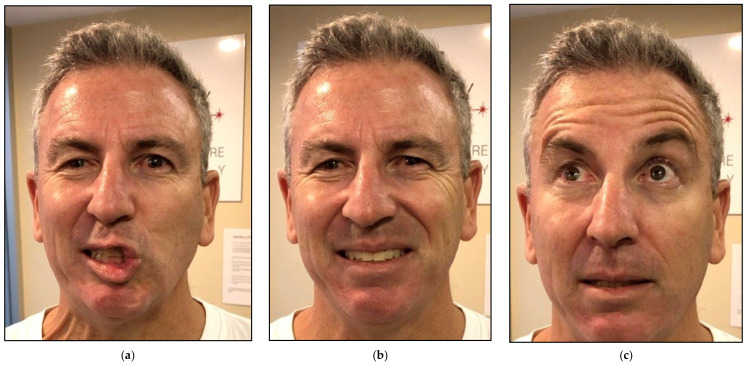
Shows a great improvement in the functionality of the facial muscles at 6 weeks post-photobiomodulation therapy. (**a**) puckering lips; (**b**) symmetrical smile; (**c**) raising eyebrows.

**Figure 12:**
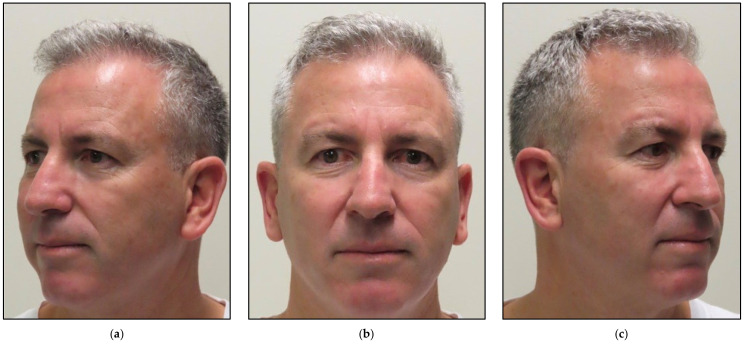
Shows improvement in the facial muscles’ functionality 20 weeks after photobiomodulation (PBM) therapy compared to Figure 8 at the initial presentation prior to PBM. (**a**) lateral view shows a significant improvement when the patient was smiling and making facial movements; (**b**) frontal view shows facial asymmetry related to paralysis of the right side of the face; (**c**) lateral view shows lack of drooping of the right corner of the mouth and a great improvement of facial symmetry.

Figure 10 shows clinical improvement of the facial muscles over the course of PBM therapy, with “18” being the maximum value and “3” being the minimal value based on HBGS. Figure 11 shows the results graphically, whereas Figure 12 shows the almost normal symmetry of the facial muscles over the course of PBM therapy at 20 weeks after PBM, indicating the recovery of the branches of the 7th cranial nerve and regression of facial paralysis.

## 4. Discussion

Our data significantly confirmed the therapeutic effects of pulsed 1064 nm PBM in restoring the functionality of the facial nerve in patients with moderate-to-severe PFP.

It is known that CCO induces an inflammatory response, activating a molecular cascade marked by an increase in proinflammatory cytokines such as IL-1β, IL-6 and TNF-α, which play important roles in the etiology and continuation of neuropathic pain [47].

The results of our study indicate that PBM can affect the mitochondrial activity and bioenergetic metabolism of the cells, resulting in an improvement in the recovery of the facial nerve. This coincided with several studies suggesting that PBM reduces inflammation and pain, prevents fibrosis, and enhances wound healing and tissue regeneration [6]. Mitochondrial biogenesis helps the cell renew the mitochondrial network and consequently improve mitochondrial function, slowing down the cascade of damage caused by mitochondrial dysfunction.

To the best of the authors’ knowledge, there is no published case series yet, describing the use of pulsed 1064 nm laser PBM as a monotherapy in resolving chronic unresponsive facial paralysis in such unique cases of different origins, after scrutinizing the literature.

There are only four case reports in the literature utilizing PBM in the management of facial paralysis [1,9,11,48]. A case report conducted by Bernal Rodriguez et al., 2020 [1] concluded that utilizing a protocol of dual-wavelength (660 nm and 808 nm) PBM with 24 consecutive PBM treatment sessions was effective in treating a facial paralysis of 8 years. We equally observed in case #2 of our study, where the symptoms were severe and chronic, that utilizing the 1064 nm near infrared (NIR), targeting deep-seated tissue with 34 PBM sessions over a period of 5 months, was effective for facial paralysis recovery. Whereas, a case of a 13-year-old girl reported by Poloni et al., 2018 [11] showed that three sessions of 700 nm (NIR) PBM (100 mW output power, 100 J/cm^2^ of energy density, 28 s per point) were effective in complete recovery and absence of muscular pain, complete regression of paralysis, and improvement of speech and chewing. Another case report of a 71-year-old [9] treated with 808 nm (NIR) PBM showed it to be effective and stressed that employing therapeutic measures as early as possible can improve the prognosis.

Our results are encouraging. In the second and third cases, the functionality of facial nerve branches returned to an almost normal level. No further treatment was needed. In the first case, as we were dealing with a chronic case of facial paralysis of 12 years, the results were not as optimal as we expected; however, a significant improvement was observed. It is noteworthy that all three patients in our case series were very satisfied with the results of the treatment and testified about their life quality improvement.

PBM involves a wide range of factors that influence optimal outcomes, such as wavelength, light properties, dosage, target optical properties and beam profile [38,49]. Underdosage results in poor cellular response, but overdosage may paradoxically inhibit cell proliferation or induce apoptosis. The authors believe the success of this chronic PFP case series is essentially due to the following fundamental factors: utilizing a collimated flat-top beam profile offering uniform cross-sectional fluence and significant efficiency in penetrating the photonic energy more deeply into tissues in the red to NIR wavelength regions [37]; employing optimal laser PBM dosimetry and treatment protocols; using a 1064 nm laser (NIR wavelength), which offers deeper penetration depth of 10 mm [50] to reach the facial nerve within the superficial musculoaponeurotic system (SMAS), where its thickness throughout the face varies from 2 to 3 mm [51]; valid reproducible assessment tools; operator’s expertise in the field of PBM.

At cellular and molecular levels, PBM therapy generates the following activities: reduction in OS, modulation of ROS, an increase in the ATP synthesis, reduction in the proinflammatory cytokines and an augmentation of the dorsal root ganglion neurons regeneration by restoring mitochondrial biogenesis. This ultimately leads to facial nerve recovery.

Although our study presents limited data with no control, which makes it difficult to perform quantitative analysis, it is a matter of fact that these patients obtained significant regression in the affected areas where the standard care treatments were unsuccessful in alleviating the chronic symptoms. Hence, despite the limitations of this study, our pulsed laser PBM dosimetry and treatment protocols demonstrated to be effective over the standard care treatment and antioxidant supplements in the management of chronic facial paralysis of long-term unresponsive symptoms to the standard care treatment. Further studies with large data are warranted to validate our 1064 nm pulsed laser PBM dosimetry delivered with a flat-top beam profile and treatment protocols in the management of chronic PFP.

## 5. Conclusions

Our results proved significantly the potential of 1064 nm laser PBM with a flat-top delivery system in the management of patients with moderate-to-severe PFP unresponsive to standard treatment care. Our 1064 nm PBM laser dosimetry protocol is effective in chronic cases of PFP, and our modified treatment protocol, depending on the origin and severity of the condition, is fundamental in optimizing facial paralysis recovery and restoring neurological competency. Extensive clinical studies are warranted to validate this protocol using large data.

## Figures and Tables

**Table 1:** Shows the laser device specifications and irradiation parameters that were utilized for all three cases.

**Device Specifications**	Manufacturer	Fotona, Ljubljana, Slovenia
	Model identifier	LightWalker
	Emitters Type	Nd:YAG laser
	Laser aiming beam	532 nm
	Medical/laser class	IV
	Beam delivery system	Fibre
	Probe design	Single probe
	Beam profile	Collimated flat-top
	Beam divergence full angle	0°
**Irradiation parameters**	Wavelength	1064 nm
	Therapeutic power output	3200 mW
	Emission mode	Pulsed
	Pulse width (microseconds)	100
	Beam spot size at target (cm^2^)	6 cm^2^
	Irradiance at target (mW/cm^2^)	533 mW/cm^2^
	Energy per pulse (J)	320 mJ
	Energy (J) per spot	192 J
	Fluence (J/cm^2^) per point	32 J/cm^2^
	Duration of irradiation (s)	60 s
	Area irradiated (cm^2^)	6 cm^2^

**Table 2:** Shows the PBM treatment protocol that was utilized for case #1.

Number of irradiated points	7	
Laser–tissue distance	In contact	
Application technique	Spotting technique	
Frequency of treatment sessions time	First month	12 sessions
Time interval between treatment sessions	1st, 3rd and 5th	
Total number of treatments and duration	27 during 6 months	

**Table 3:** Improvement of Bell’s palsy using the House–Brackmann grading scale of facial muscles’ functionality.

PBM Treatment Week Number	Functionality Level	Facial Symmetry	Facial Muscles’ Functionality			Total
			Forehead	Eye	Mouth	
0	Severe	Asymmetric	5	3	5	13
1	Severe	Asymmetric	5	3	5	13
2	Severe	Asymmetric	5	3	5	13
3	Severe	Asymmetric	5	3	5	13
5	Moderate	Asymmetric	4	3	5	12
7	Moderate	Asymmetric	4	3	4	11
11	Moderate	Asymmetric	3	3	4	10
15	Moderate	Asymmetric	3	2	3	8

**Table 4:** Shows the PBM treatment protocol that was utilized for case #2.

Number of irradiated points	12	
Laser–tissue distance	In contact	
Application technique	Spotting technique	
Frequency of treatment sessions	First month	12 sessions
Time interval between treatment sessions	1st,3rd and 5th day	
Total number of treatments and duration	22 during 4 months	

**Table 5:** Shows an improvement in Lyme disease palsy using the House–Brackmann grading scale.

PBM Treatment Week Number	Functionality Level	Facial Symmetry	Facial Muscles’ Functionality			Total
			Forehead	Eye	Mouth	
0	Paralysis	Asymmetric	6	6	6	18
1	Severe	Asymmetric	6	6	5	17
2	Severe	Asymmetric	5	5	4	14
3	Moderate	Asymmetric	4	4	3	11
4	Moderate	Asymmetric	3	4	3	10
5	Moderate	Asymmetric	3	4	2	9
8	Mild	Mildly asymmetric	2	4	2	8
9	Almost normal	Mildly asymmetric	2	3	2	7
13	Almost normal	Symmetric	2	2	2	6
23	Normal	Symmetric	1	1	1	3

**Table 6:** Shows the PBM protocol that was utilized for case #3.

Number of irradiated points	11	
Laser–tissue distance	In contact	
Application technique	Spotting technique	
Frequency of treatment sessions time interval and duration	First month	22
	Second month	11
	Third month	7
	Fourth month	4
	Fifth month	4
Total number of treatments	48	

## Data Availability

All the data are available in the text.
